# Assessing effects of personal behaviors and environmental exposure on asthma episodes: a diary-based approach

**DOI:** 10.1186/s12890-019-0998-0

**Published:** 2019-12-02

**Authors:** Ta-Chien Chan, Tsuey-Hwa Hu, Yen-Hua Chu, Jing-Shiang Hwang

**Affiliations:** 1 Research Center for Humanities and Social Sciences, Academia Sinica, 128 Academia Road, Section 2, Taipei, Taiwan; 2 Institute of Statistical Science, Academia Sinica, 128 Academia Road, Section 2, Taipei, Taiwan

**Keywords:** Asthma, Diet behavior, Air pollution, Weather, Exercise, Smoke, Perception

## Abstract

**Background:**

Quantifying the effects of personal health behaviors and environmental exposure on asthma flare-ups is a challenge. Most studies have focused on monitoring the symptoms and drug usage for relieving symptoms. In this study, we emphasize the need to understand how personal and environmental conditions are related to the occurrence of asthma symptoms.

**Methods:**

We designed an online health diary platform to collect personal health behaviors from children, their parents and other adults with any allergic diseases including asthma, allergic rhinitis, atopic dermatitis and allergic conjunctivitis. The participants used mobile devices or computers to record their daily health-related activities such as sleep, exercise, diet, perception of air quality and temperature, and asthma symptoms. The participants also recorded secondhand smoke exposure and the time of activities, which were combined with ambient air quality measurements for calculating personal air pollution exposure. A generalized linear mixed model was used to estimate the effects of the factors.

**Results:**

During the study period (January 2017–June 2017, and October 2017–September 2018), 132 participants provided 25,016 diary entries, and 84 participants had experienced asthma symptoms in 1458 diary entries. The results showed some different risk factors for the minors and adults. For minors, high-intensity exercise, contact with persons with influenza-like illness (ILI) and the perception of hot temperature and bad indoor air quality were associated with the occurrence of asthma episodes. The identified risk factors for the adult participants included having dehumidifiers at home, exposure to secondhand smoke, having bad sleep quality, contact with persons with ILI, not eating fruit and seafood, perceiving cold temperature, bad quality of indoor and outdoor air, and exposure to high concentration of ozone.

**Conclusions:**

The revealed personal risk factors and perceptions of air quality and temperature may provide guidance on behavioral change for people susceptible to asthma to help control acute onset and severe exacerbation of asthma flare-ups.

## Background

Asthma is the most prevalent chronic respiratory disease, and causes high disease burden, not only for children but also for adults. As of 2015, the overall asthma age-standardized mortality worldwide had declined by 58.8% since 1990, with a 95% confidence interval (CI) of (39.0, 69.0%). However, the prevalence of asthma had increased by 12.6% (9.0, 16.4%) [1]. In 2016, asthma for all ages contributed to 23.7 million disability-adjusted life years globally [2]. Although the development of asthma can be attributed to genetic, environmental and host factors [3], prevention of the disease’s onset and relapse is important to public health. For asthma patients, good control of asthma is highly related to their quality of life [4], reduces medical costs [5], and prevents further exacerbation [6]. In addition to the pharmaceutical approach to symptom control such as using inhaled corticosteroids (ICS), non-pharmacological strategies such as physical activity and weight loss are also very important [7].

In previous studies, asthma relapse was attributed to personal and environmental factors. Some patients did not manage their symptoms well and went to the emergency department frequently [8]. In addition, female adults and previous ICS usage were also found to be significantly associated with relapse occurrence [9]. Exposure to secondhand smoke [10] and air pollution [11, 12] also play important roles in elevating the risk of asthma medical visits. However, if we only observe the relationship between demographic and environmental factors and medical visits, we cannot know the exact personal risk factors for prevention because the medical records don’t have that information. For example, diet behaviors [13], exercise [14], and sleeping quality [15] are reported to be correlated with asthma relapse. All this information is dynamic in daily life and not easy to capture in traditional one-shot surveys. The diary approach might be a good solution to collect health behaviors and estimate environmental exposure levels. It also can help participants reduce recall bias. However, in the current literature, research using asthma-related diaries has focused more on measuring the scores of asthma symptoms and medication usage [16, 17]. The dynamic information of daily activity and exposure was not collected for assessing the risk factors and the occurrence of asthma symptoms.

In this study, we designed a diary-based online survey to collect personal health behaviors, perceptions of environmental conditions, and environmental exposure for each participant. We have applied this diary approach successfully to track mood changes with health behaviors [18], clarify how personal mood occurs concurrently among network members [19] and examine influenza-like illness (ILI) contagion through social contact networks [20]. The aim of this study is to elucidate the conditions leading to flaring up of asthma symptoms through a prospective long-term follow-up using online diary data. Through this approach, we can quantify and differentiate the risks from the personal and environmental levels and provide advice to patients susceptible to asthma to reduce the chance of attacks.

## Methods

### Ethics

This study was approved by the Institutional Review Board on Biomedical Science Research, Academia Sinica (AS-IRB-BM-16058 v.2). For participants under the age of 18, the research team received written consent from their parents or legal guardians before activating their accounts. The health diary data for analysis were stripped of personal identification information, which was replaced with a serial number to protect participants’ privacy.

### Health diary program

We designed an online platform named “Health Diary” to collect participants’ daily health behaviors, perception of environmental conditions, exposure to allergens, contacts with persons with ILI, indoor and outdoor exposure time, and self-report of any uncomfortable symptoms including asthma-related symptoms. The platform utilized a responsive web design to facilitate filling in the questionnaire through computer, mobile phone or tablet. Upon enrollment, participants were asked to fill in their demographic data including gender, age, township of residence, whether they had dehumidifiers at home, whether they had carpets at home, whether they had black mold spots on the walls, and whether they had been clinically diagnosed with asthma, allergic rhinitis, allergic conjunctivitis, or atopic dermatitis.

In sleeping behavior, the diary questionnaire items included what time they got up and went to bed, and sleeping quality (very good, good, fair, poor, very poor). Among food intake, we listed 10 categories of food including (1) whole grains, rootstock, rice and flour, (2) vegetables, (3) fruits, (4) meat, (5) seafood, (6) legumes, (7) eggs, (8) dairy products, (9) fried foods, and (10) desserts and sugary drinks. The portions of the food were measured on five-point scales (i.e. 0, 0.5, 1, 2, 3+) with different units. Each food category on the online questionnaire item was accompanied by an explanation to ensure all participants had the same understanding of the size of a portion. The participants recorded both exercise intensity and time. In health-related status, the participants reported their overall mood during the past day, and any uncomfortable symptoms such as breathing difficulty or wheezing, persistent cough without catching a cold, pain in the throat, chest pain, sneezing, nasal congestion, itchy eyes or skin, fever (> 38 °C), clinically diagnosed cold, encountering persons with ILI symptoms, and using ICS or a bronchodilator. Environment-related items in the diary included experience of secondhand exposure in the working space or at home, whether they played with hairy pets, whether they saw cockroaches at home, the types and time duration of transportation, how long they stayed indoors, the perceptions of temperature (very cold, cold, fair, hot, or very hot), and outdoor/indoor air quality (very bad, bad, fair, good, or very good), which was grouped into two categories of bad air quality (very bad, bad) and the others in the analysis.

### Participant recruiting

The recruitment of participants was from two channels. The first was from a nationwide survey of allergic diseases conducted by Dr. Chi-Hsin Chen’s research team at National Taiwan University Hospital. We sent an invitation letter to students from the fifth to ninth grade who were confirmed to have or were considered susceptible to asthma in the survey, and their parents, to join this project. The second channel was from a participatory cohort of our previous study [18]. We invited the participants in that study who had asthma, allergic rhinitis, allergic conjunctivitis, or atopic dermatitis to join this project. The participants were informed that they would record an online diary at least twice a week and take a few minutes to complete a diary. Based on our visiting logs of the website, the participants spent an average of 3 min per day to complete the diary. To encourage long-term participation, we gave a small reward each month to the participants who provided diary data of good quality at least twice a week.

### Weather and air pollution data

At the baseline, we had already collected the township of residence for each participant. Therefore, we used that geographic information to locate the nearest ambient air quality monitoring stations operated by the Taiwan Environmental Protection Agency (TEPA). The open data of air quality and weather can be downloaded from the TEPA website (https://taqm.epa.gov.tw). The hourly air pollutants including PM_2.5_ and O_3_ and the hourly temperature and relative humidity (RH) were used for computing daily concentrations of the 24-h average for PM_2.5_ and maximum 8-h average for O_3_, temperature difference and average RH.

### Study period and design

In the first wave of the study from January 1, 2017 to June 30, 2017 we collected online diaries. Preliminary results from the first-wave data showed some interesting findings which needed to be further verified and confirmed. We then extended the study for one more year, from October 1, 2017 to September 30, 2018 by asking the participants to continue and inviting new subjects to participate. During the studied period, there were 219 participants registered our program. However, 27 participants did not complete the baseline questionnaires, or did not have any allergic diseases defined in this study. Therefore, we removed those people because of ineligibility. Among the 192 qualified participants, we set up an indicator to filter out participants with a low response rate. The participants to be included should stay in the program at least 30 days and fill in the health diaries at least 8 days. Among the 122 adult participants, 97 were selected for the study. For the 70 minors, 35 participants were selected for the analysis. We made a comparison of basic characteristics between the included participants and the others (Appendix 1). The comparison results showed that most of the characteristics are similar except for the proportion of males, which is slightly higher in the included minors. The remaining data for analysis consisted of 25,016 health diary entries from 132 participants.

The asthma symptoms here are defined as breathing difficulty and wheezing or persistent cough without catching cold. We adopted a case-crossover design to define onset episode and control episode. The case episode of having asthma symptoms is defined as the first day of reporting defined symptoms and without any defined symptoms in the previous 3 days. With the definition, we identified 489 diary entries recorded by 84 participants during the study period as case episodes. The control episode without asthma symptoms can be classified into two types. The first type is the absence of any asthma symptoms during the 3 days before and 3 days after the case day. This definition is for those having asthma symptoms during the study period. The reason why we use 3 days as the observation window is that we asked the participants to fill in at least two diary entries per week. Our data showed that nearly 89% of diary entries accompanied another one from the same participant on the day 3 days earlier. In addition, an asthma flare-up may last from a few hours to days. We also found that for nearly 74% of the asthma flare-up days, the participant also reported asthma symptoms on at least one of the previous 3 days. The second type is lack of any asthma symptoms during the entire study period. Based on the above rules of selecting controls, we extracted 22,864 diary entries as control episodes.

### Statistical analysis

The response variable is binary, with 1 representing case episode and 0 representing control episode. We first used a logistic regression with a stepwise variable selection procedure to find the best explanatory variables according to the Akaike information criterion (AIC). Four categories of potential explanatory variables were considered for model selection including the baseline covariates, diary-related variables, environmental exposures, and perception of environmental conditions. The baseline covariates included gender, asthma history, dehumidifiers at home and having black mold at home. The diary-related variables included secondhand smoking exposure, touching hairy pets or seeing cockroaches at home, sleep quality, contact with persons with ILI symptoms, metabolic equivalent, foods (cereal, vegetables, fruit, meat, seafood, fried food, dairy, dessert, beans, and eggs). For environmental exposure, we considered daily temperature difference, average relative humidity, outdoor exposure to PM_2.5_ and ozone. For personal perception, we explored feelings of temperature and quality of indoor and outdoor air. Then, we used a generalized linear mixed model (GLMM) with the selected explanatory variables from the stepwise logistic regression model and considered the random effects for the individuals in the model to examine how health behaviors, perception of environmental conditions, and environmental exposure were associated with the flare-up of asthma symptoms. Since the overall levels of the pollutants were quite different among the living areas of the participants and time spent outdoors also varied a lot for each individual during the study period, we proposed using the time-space-standardized level to represent outdoor exposure to the pollutants. The time-space-standardized exposure level to a pollutant is calculated by (*P*_*ij*_ − *A*_*i*_) × *T*_*ij*_/24, where *P*_*ij*_ represents daily concentration of the pollutant on day *t*_*ij*_ from the ambient air quality monitoring station nearest to the i^th^ participant’s residence, *A*_*i*_ represents the average concentration of the pollutant during the study period from the same station, and *T*_*ij*_ represents the total hours the subject spent outdoors on day *t*_*ij*_. Random intercept representing variation of risks among the individuals not explained by the covariates was included in the GLMM model. The above modeling procedures were applied to analyze data of 35 minors (age < 18) and 97 adults, separately. We used R software (R Foundation for Statistical Computing, Vienna, Austria; version 3.5.1) [21] and the “glm” function to do stepwise logistic regression, and R package “lme4” [22] using the “glmer” function to estimate the parameters in the final GLMM.

## Results

The results reported here were based on the analysis of 25,016 records provided by the 132 qualified participants. During the study period, 84 participants reported asthma symptoms in 1458 diary entries. Thirty-five minors aged from 10 to 17 contributed 5608 diary entries, and 97 adults aged 18 to 73 contributed 19,408 entries (Table 1). Among the 132 participants, females accounted for 56%, while the minor group had more male participants (66%). More than half of the participants (64%) had at least one episode with asthma symptoms during the study period. Our baseline survey shows that 89% of participants had a history of allergic rhinitis. Asthma history was higher among younger participants (40%).

**Table 1:** Summary of demographic information for 132 participants

Age groups	N	Male (%)	Number of diary entries	Asthma symptoms during study	Past allergic history			
					Asthma	Allergic rhinitis	Allergic conjunctivitis	Atopic dermatitis
10–17	35	23 (66%)	5608	20 (57%)	40%	97%	37%	37%
18–73	97	35 (36%)	19,408	64 (66%)	12%	86%	38%	33%
10–73	132	58 (44%)	25,016	84 (64%)	20%	89%	38%	34%

In Table 2, we computed the frequency of the identified explanatory variables from the stepwise logistic regression model to understand the occurrence of those risk factors. Some factors had a higher proportion in adults, including dehumidifier use at home (69.1%), touching hairy pets or seeing cockroaches at home (24.8%), secondhand smoking exposure (18.1%), contact with persons with ILI symptoms (14.2%), having poor sleep quality (9.7%), and perception of hot temperature (31.1%). Some factors had a higher proportion in minors, including perception of very hot temperature (19.4%), bad quality of indoor air (13.2%) and outdoor air (21.9%), and doing high-intensity physical exercise (MET> 0 and < =8: 47.1%). The distribution of intake of the food items is given in Table 3. The comparison between minors and adults in the median of the food portions was very similar, except for 0.5 portions higher of seafood in adults and 0.5 portions higher of dairy products in minors.

**Table 2:** Summary of the explanatory variables retained in the stepwise logistic regression model in terms of number of participants for the time-independent variable and diary entries for the time-dependent variables

Variables	Frequency (%) Minors	Frequency (%) Adults
*Time-independent*		
Dehumidifiers at home	20 (57.1%)	67 (69.1%)
More than one wall having black molds at home	6 (17.1%)	16 (16.5%)
*Time-dependent*		
Touch hairy pets or see cockroaches at home	1159 (20.7%)	4820 (24.8%)
Secondhand smoking exposure	653 (11.6%)	3508 (18.1%)
Contact persons with ILI symptoms	658 (12.0%)	2707 (14.2%)
Poor sleeping quality	231 (4.1%)	1880 (9.7%)
Perception of temperature		
very cold	277 (4.9%)	893 (4.6%)
cold	996 (17.8%)	3536 (18.2%)
fair	1689 (30.1%)	5974 (30.8%)
hot	1560 (27.8%)	6040 (31.1%)
very hot	1086 (19.4%)	2965 (15.3%)
Perception of bad indoor air quality	742 (13.2%)	1965 (10.1%)
Perception of bad outdoor air quality	5466 (21.9%)	4063 (20.9%)
Metabolic equivalent (MET)		
0	2852 (50.9%)	12,018 (61.9%)
> 0 and ≤ 8	2639 (47.1%)	7361 (37.9%)
> 8	117 (2.1%)	29 (0.1%)

**Table 3:** The distribution of portions of the food intakes before model selection

	Food	Min	Q1	Median	Q3	Max
Minors	Whole grains, rootstock, rice and flour	0	2	2	3+	3+
	Vegetables	0	1	2	3+	3+
	Fruit	0	1	1	2	3+
	Meat	0	1	1	2	3+
	Beans	0	0	0.5	1	3+
	Seafood	0	0	0	1	3+
	Fried food	0	0	0	1	3+
	Dessert	0	0	1	1	3+
	Eggs	0	0	1	1	3+
	Dairy products	0	0	1	1	3+
Adults	Whole grains, rootstock, rice and flour	0	2	2	3+	3+
	Vegetables	0	1	2	3+	3+
	Fruit	0	0.5	1	2	3+
	Meat	0	1	1	2	3+
	Beans	0	0	0.5	1	3+
	Seafood	0	0	0.5	1	3+
	Fried food	0	0	0	1	3+
	Dessert	0	0	1	1	3+
	Eggs	0	0	1	1	3+
	Dairy products	0	0	0.5	1	3+

In Table 4, we listed the summarized statistics of the 25th percentile (Q1), median, 75th percentile (Q3), mean and standard deviation for environmental variables measured at the monitoring stations nearest to the participants during the study period. The average daily temperature difference was 6.04 °C, the average RH was 74.24%, the average PM_2.5_ was 21.85 μg/m^3^ and average of the maximum 8-h average O_3_ concentration was 44.76 ppb.

**Table 4:** The summarized statistics of weather conditions and concentrations of air pollutants during the study period

Variables	Q1	Median	Q3	Mean	SD
Daily temperature difference (°C)	4.15	6.00	7.81	6.04	2.47
Relative humidity (RH) (%)	68.56	74.46	80.29	74.24	9.10
PM_2.5_ (μg/m^3^)	12.39	19.67	29.00	21.85	12.06
Max 8-h average O_3_ level (ppb)	34.12	43.25	54.38	44.76	15.33

The estimated results from the generalized linear mixed model showed effects of different risk factors for the minors and adults. Table 5 summaries estimated odds ratios of influential factors in the GLMM. The OR of asthma episodes for young participants having contact with persons with ILI symptoms versus no contact was 2.64 with 95% CI = (1.48, 4.70). The odds ratios for the minors with high-intensity exercise (MET > 8), perceiving hot temperature and bad indoor air were 5.45 (2.11, 14.06), 1.82 (1.00, 3.31) and 2.15 (1.08, 4.26), respectively. For adults, more risk factors associated with the occurrence of asthma episodes were identified from the model. Adult participants with dehumidifiers at home had a higher OR of 2.42 (1.12, 5.20). The odds ratios for adults exposed to secondhand smoke, having bad sleep quality and contact with persons with ILI symptoms were 2.24 (1.61, 3.10), 1.85 (1.30, 2.62), and 2.02 (1.50, 2.72), respectively. The perception of very cold, cold temperature, bad indoor air and outdoor air quality were all associated with the episodes with OR = 1.96 (1.23, 3.14), 1.46 (1.07, 1.99), 1.50 (1.01, 2.24) and 1.38 (0.99–1.93), respectively. We also found that time-space-standardized ozone level was significantly associated with the probability of asthma episodes’ occurrence for the adult participants with a coefficient estimate of 0.06 (0.01, 0.11). In addition, there were some factors showing protective effects against triggering asthma episodes such as being male, eating more fruits and seafood, and perception of very hot temperature for adults.

**Table 5:** The estimated odds ratios of influential risk factors identified in the generalized linear mixed models for the minors and adults

Variables	Minors			Adults		
	OR	95% CI		OR	95% CI	
Male	1.82	0.25	13.33	0.37	0.18	0.77
Dehumidifiers at home	1.06	0.15	7.50	2.42	1.12	5.20
Secondhand smoking exposure	–	–	–	2.24	1.61	3.10
Poor sleeping quality	–	–	–	1.85	1.30	2.62
Contact with persons with ILI	2.64	1.48	4.70	2.02	1.50	2.72
Metabolic equivalent (MET, 0 as reference)						
> 0 and < =8	1.11	0.68	1.81	0.78	0.58	1.06
> 8	5.45	2.11	14.06	7.26	0.82	64.02
Fruit (> = 0 and < =0.5 portion as reference)						
> 0.5 and < =2	–	–	–	0.61	0.45	0.82
> 2 and < =3+	–	–	–	0.50	0.27	0.92
Seafood (0 portion as reference)						
> 0 and < =1	0.94	0.59	1.49	1.10	0.85	1.44
> 1 and < =3+	0.67	0.22	1.99	0.44	0.24	0.79
Perception of temperature (fair as reference)						
very cold	2.35	0.94	5.88	1.96	1.23	3.14
cold	1.55	0.77	3.12	1.46	1.07	1.99
hot	1.82	1.00	3.31	0.77	0.56	1.05
very hot	0.95	0.45	2.01	0.37	0.23	0.59
Perception of bad indoor air	2.15	1.08	4.26	1.50	1.01	2.24
Perception of bad outdoor air	–	–	–	1.38	0.99	1.93

## Discussion

Through this prospective study, we successfully captured 1458 episodes of having the defined asthma symptoms. The study finds an acute effect of ambient ozone exposure on asthma symptoms. In addition, health behaviors and perceptions of the temperature, as well as indoor and outdoor air quality, are all shown to be related to the occurrence of asthma symptoms, and turn out to be critical information. It is not easy to collect these dynamic data over time, and such an approach is missing in the literature. Here, we used a diary-based approach to record time-varying health behaviors and exposures. This can help us understand what conditions will result in a higher chance of having asthma symptoms in our daily life and suggest preventive measures for reducing the risk. In the following three paragraphs, we will discuss our findings on personal risk factors, perceptions of environmental conditions and air pollutants.

Personal risk factors are what we do and encounter in daily life. The first risk factor is smoking. One Korean community survey [23] found that former and current smoking and passive smoke exposure are positively correlated with the occurrence of asthma symptoms such as wheezing, and wheezing during exercise. In addition, longer exposure to secondhand smoke (> = 1 h/day) showed a higher risk than shorter exposure time (< 1 h/day). The measured interval of their survey was one to 2 years. The mean OR for having wheezing symptoms under passive smoke exposure at home (> = 1 h/day) was 1.63 and that at work (> = 1 h/day) was 1.51. Based on our findings, the OR of secondhand smoke exposure in adults is 2.24 higher than theirs. The reason may be that our measurement was daily and can reflect more episodes and reduce the recall bias from a long follow-up period. The second risk factor is contact with persons with ILI symptoms. The underlying assumption is that persons with asthma after contact with persons with ILI symptoms might get infected, leading to asthma attacks [24]. Patients with asthma have increased expression of high-affinity IgE receptors (FcεRI) on plasmacytoid dendritic cells (pDCs), and one study found that activation of FcεRI in vivo may result in reduced innate immunity recognition of response to influenza virus [25]. From our indirect findings, contact with persons with ILI not only reflected direct transmission from the contact persons but also represented a possible influenza epidemic in their surroundings. Therefore, the allergic participants would have a higher chance of induced asthma symptoms. This risk factor is significantly identified in our analysis for both minors and adults.

The third risk factor is high intensity of exercise. Although the recent clinical guideline is to encourage moderate exercise for asthmatic children and adolescents [26], and one study also suggested that high-intensity exercise can help control moderate to severe asthma [27], some asthma patients are not well trained or not aware of the risk of exercise-induced asthma. In our finding, we found that those minors who exercise at MET level > 8, which is equivalent to running and rope jumping, have higher risk of induced asthma symptoms. The fourth risk factor is diet behavior. According to one multicentric study in 20 countries [28], higher frequent consumption of fruit, vegetables, and fish was associated with lower prevalence of current wheezing. Their findings are consistent with our study, especially for the adults.

Actual air pollution is included in the questionnaire of the Asthma Trigger Inventory, which lists six factors triggering asthma, including psychology, animal allergens, pollen allergens, physical activity, infection, and air pollution [29]. We included ambient measures in temperature, relative humidity and air pollution to quantify their effect on asthma attacks, but temperature was not significant in the first stage of variable selection, probably because the measurement was quite coarse in spatial resolution and also was quite different for each person. We also included the real feeling or perception of temperature and air pollution, and these turned out to have significant effects, most likely because they are more direct measurements for the individual participants. In our findings, the perceptions of cold temperature and bad indoor and outdoor air quality are significant factors for having asthma symptoms. And hot temperature (as measured through perceived temperature) may have protective effect for adults but a risk for minors.

In most studies, researchers have usually utilized second-hand morbidity data and ambient environmental data to model their relationships. The diary approach in this study can help track the real perception of the respondents on environmental conditions which might be used as a proxy of personal exposure to temperature and air pollutants. Temperature, either hot or cold, was found to have effects on triggering asthma attack [30, 31]. The biological mechanism on how high and low temperature affect asthma was well reported by Xu et al. [31]. In summary, high temperature can activate vagal bronchopulmonary C-fiber sensory nerves related to reflex bronchoconstriction, enhance the growth of and exposure to indoor aeroallergens, and expose people to high level of air pollutants. Low temperature is related to decreased lung function and lower lung capacity, a suppressed immune system, a higher chance of transmission of respiratory virus, inflammation of the airways, and production of the mucin protein which triggers asthma attack. Therefore, asthma patients are sensitive to the temperature. This study used perception to capture personal sensitivity to changing temperature and air quality. The results of high association between personal perception of environment conditions and asthma episodes in daily life provide self-protective guidance for asthma patients. Asthma patients should not only pay attention to the forecast of temperature and air quality but also watch out for their subjective feeling of the environmental conditions to prevent relapse. In addition, we also validated the relationship between actual measurement and participants’ perception. There were three questions related to participants’ perception including ambient temperature, and quality of indoor and outdoor air. In the actual measurement, we can only have ambient temperature and outdoor air pollution such as O_3_ and PM_2.5_. Thus, we examined the relationship between actual outdoor measurements and their perceptions in Appendix 2. The results showed the trend of perception is consistent with the actual measurement.

One study used personal monitoring equipment to collect the breathing zone exposure to PM_10_ and PM_2.5_ [32]. They found higher personal exposure to PM_10_ is inversely correlated with asthma control and health-related quality of life. From one global estimation, 9–23 million and 5–10 million annual asthma emergency room visits globally in 2015 could be attributable to ozone and PM_2.5_, respectively [33]. Another study, in China, reported that parents’ perception of air quality and relative humidity are significantly correlated with children’s allergic diseases including asthma and wheezing [34]. These all support the notion that personal exposure and perception are highly correlated with asthma control. An acute impact of ambient air pollution on acute asthma attack has been reported by many studies [35, 36]. However, the actual exposure time and the indoor and outdoor exposure time were mostly unavailable. We used the diary approach to let our participants record their exposure time. Thus, we can use the time-space-standardized exposure measurement to more accurately identify air pollutants’ effect. In our study, we found variation of ozone is correlated with asthma symptoms among the adults, while changes in outdoor PM_2.5_ exposure have no significant correlations.

There are two limitations in this study. The first one is the limited sample size. Because of the nature of our study design, the participants needed to fill in the diary for many months. It is hard to keep a large number of participants for such a long time. Due to the limitation of small sample size and voluntary-based participation, we cannot generalize our findings to the general population. This limitation is quite similar to that for another asthma daily symptom diary [37]. The second one is the confirmation of asthma patients. Our student participants were mostly identified through a nationwide survey and had a higher percentage of diagnosed asthma than our adult participants. For the adult participants, the percentage of confirmed asthma patients was low but they each had at least one self-reported allergic disease in our inclusion criteria. Those with allergic disease or who are susceptible to asthma all have high potential to develop asthma. In this study, we used the two common asthma symptoms as our major outcomes and identified the relationship between risk factors and the onset of symptoms.

## Conclusions

Our observations through diary-based follow-up find that exposure to secondhand smoke, persons with ILI, and high concentrations of ozone were linked to higher chances of having asthma episodes. Keeping good diet behaviors and sleeping quality, and paying more attention to personal perception of temperature and air quality might reduce the chance of asthma episodes. These behavioral changes may help improve asthma control, preventing acute onset and severe exacerbation of asthma flare-ups.

## Figures and Tables

**Fig. 1:**
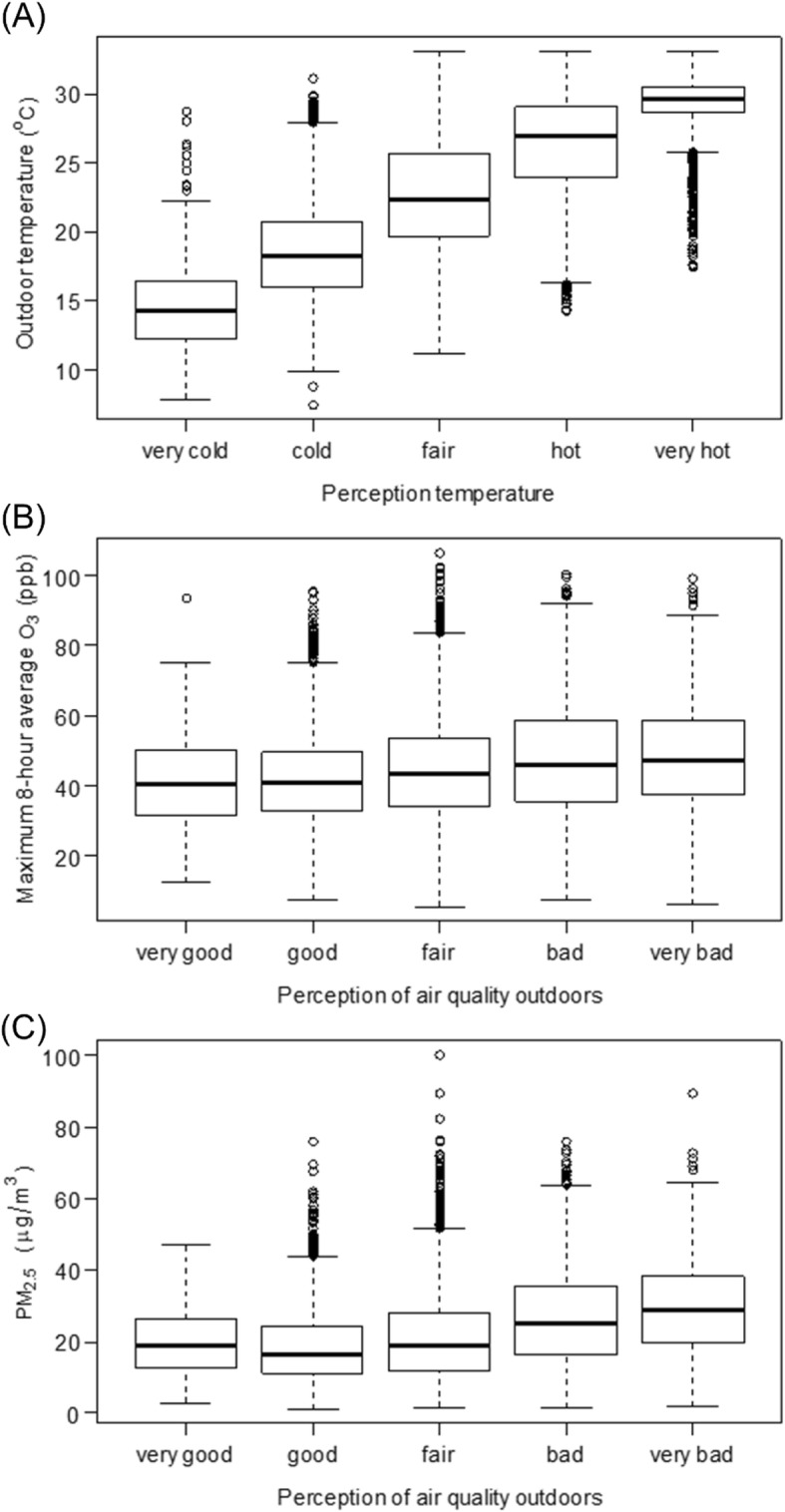
The relationship between participants’ perceptions and actual measurement (A) ambient temperature (°C) (B) O_3_ (ppb) (C)PM_2.5_ (μg/m^3^)

## Data Availability

The datasets used and/or analysed during the current study are available from the corresponding author on reasonable request.

## Appendix 1

**Table 6:** The comparison of baseline characteristics of the 132 participants included for analysis and the others

Baseline characteristics	Minors		Adults	
	Included	Excluded	Included	Excluded
Sample size	35	35	97	25
Proportion of males	0.66	0.51	0.36	0.36
Average age	12	13	41	42
Proportion of *k* allergic diseases				
*k* = 1	0.34	0.34	0.51	0.52
*k* = 2	0.31	0.37	0.35	0.40
*k* = 3	0.23	0.23	0.09	0.08
*k* = 4	0.11	0.06	0.05	0.00

## Abbreviations

FcεRI: High-affinity IgE receptors
GLMM: Generalized linear mixed model
ICS: Inhaled corticosteroids
ILI: Influenza-like illness
MET: Metabolic equivalent
pDCs: Plasmacytoid dendritic cells
RH: Relative humidity
TEPA: Taiwan Environmental Protection Agency
